# Precise diagnosis and targeted therapy of nodal T-follicular helper cell lymphoma (T-FHCL)

**DOI:** 10.3389/fonc.2023.1163190

**Published:** 2023-04-28

**Authors:** Jun Du, Shikai Jin, Minghui Zhang, Xuehang Fu, Jingwen Yang, Liwen Zhang, Zhenwei Chen, Zoufang Huang, Weisong Li, Jian Hou, Ting Wang

**Affiliations:** ^1^ Department of Hematology, Renji Hospital, School of Medicine, Shanghai Jiao Tong University, Shanghai, China; ^2^ Department of Clinical Medicine, Shanghai Jiao Tong University School of Medicine, Shanghai, China; ^3^ Department of Hematology, The First Affiliated Hospital of Gannan Medical University, Ganzhou, China; ^4^ Department of Pathology, The First Affiliated Hospital of Gannan Medical University, Ganzhou, China

**Keywords:** nodal T-follicular helper cell lymphoma, PTCL, AITL, TFH, targeted therapy

## Abstract

Nodal T-follicular helper cell lymphoma (T-FHCL) derived from T-follicular helper (Tfh) cell falls into a heterogeneous category of peripheral T-cell lymphoma (PTCL). Due to the limited number of therapeutic regimens and limited first-line efficacy, T-FHCL has a poor prognosis, and there is an urgent need for effective targeted therapies. With advancements in sequencing technologies, especially single-cell sequencing and next-generation sequencing, more specific genetic aberrations characteristic of T-FHCL can be discovered, allowing for precise molecular diagnosis and specific research on novel agents. Many biomarker-targeting agents, used either alone or in combination, have been tested, and they have generally enhanced the therapeutic outcomes of T-FHCL. Histone deacetylase inhibitors achieve significant clinical benefits in the treatment of T-FHCL, especially in combination therapy. Chimeric antigen receptor T-cell (CAR-T-cell) immunotherapies, hematopoietic stem cell transplantation, and other potential agents merit further study.

## 1 Introduction

Peripheral T-cell lymphomas (PTCLs) are a heterogeneous and aggressive group of non-Hodgkin lymphomas (NHLs), accounting for 5%-20% of NHLs worldwide (1). Mature T-cell lymphoma is classified into 30 subtypes, such as peripheral T-cell lymphoma not otherwise specified (PTCL-NOS) and adult T-cell leukemia/lymphoma (ATLL), in the 2022 revised 5th edition of the World Health Organization (WHO) classification of hematolymphoid tumors: lymphoid neoplasms (2).

The first-line chemotherapy for PTCL is the CHOP (cyclophosphamide, doxorubicin, vincristine, and prednisone) regimen, based on experience with aggressive B-cell lymphomas (3). However, due to the heterogeneity of molecular pathogenesis, the CHOP regimen has limited efficacy for PTCL. Etoposide plus CHOP (CHOEP) resulted in moderately better outcomes in higher-risk younger patients (4). In a retrospective analysis assessing 906 cases of PTCL, the CHOEP regimen resulted in a superior prognosis compared to CHOP in terms of 5-year progression-free survival (PFS) (59.0% *vs.* 32.9%) and 5-year overall survival (OS) (65.6% *vs.* 47.6%) (4). Even so, the efficacy of the CHOP and CHOEP regimens can be improved through combining targeted agents, such as brentuximab vedotin, alemtuzumab and lenalidomide.

Hematopoietic stem cell transplantation (HSCT) is generally a consolidation therapy in first-line treatment. In the first complete remission, high-dose chemotherapy followed by autologous stem cell transplantation (auto-SCT) improved OS and PFS in AITL but not in other PTCL subtypes (5). Nevertheless, prospective and randomized trials are lacking on account of aggressiveness and intensity. Therefore, the broader applicability of auto-SCT is difficult to determine. Furthermore, assuming that auto-SCT is unavailable due to stem cell mobilization failure, allogeneic stem cell transplantation (allo-SCT) remains a possible alternative as salvage therapy. Multiple studies have shown that auto-SCT in the first remission and allo-SCT in relapsed disease provide mediocre benefits over chemotherapy alone for patients with PTCL with sufficient disease control (6).

With advancements in molecular pathology, novel targeted agents were considered promising for PTCL. Many targeted agents have been applied in PTCL, such as monoclonal antibodies, histone deacetylase inhibitors (HDACis), and phosphatidylinositol 3-kinase inhibitors (PI3Kis). However, the effectiveness and safety of many of these agents *in vivo* remain to be determined, and more clinical trials are needed for verification. In addition, the subtypes of PTCL respond differently to the same agent due to diverse genetic aberrations. Hence, in this review, we concentrate on a specific subgroup of PTCL, nodal T-follicular helper cell lymphoma (T-FHCL), and discuss its therapeutic responses to different targeted agents.

**Figure 1:**
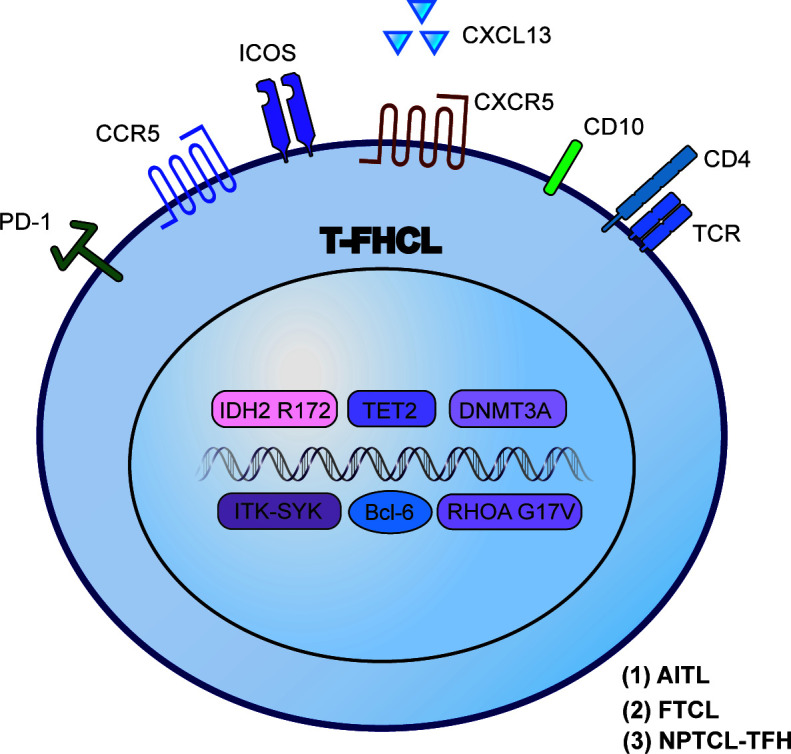
Characteristic biomarkers and genetic aberrations in T-FHCL. Identification of Tfh markers, epigenetic mutations and other specific aberrations aids the precise diagnosis of T-FHCL at the molecular level.

T-FHCL was reclassified as a new umbrella category of PTCL in 2017, which continues to be used in 2022 (2). To support its Tfh lineage, the presentation of at least two but ideally three or more Tfh markers, such as Bcl-6, CD10, PD-1 (CD279), ICOS, CXCR5, CXCL13, CCR5, SAP, MAF, and CD200, is recommended (7). In addition, the most sensitive markers are PD-1 and ICOS, while the most specific markers are CD10 and CXCL13 (7). Based on differences in clinical, histological, immunophenotypic, cytogenetics and molecular features, T-FHCL can be subdivided into three subtypes: angioimmunoblastic-type, follicular-type, and not otherwise specified. To avoid ambiguity, we adopted the nomenclature of the 2017 revised classification in this review, described below (**Table 1**; **Figure 1**).

**Table 1:** The characteristics of three entities in T-FHCL (1, 7–18).

	AITL	FTCL	NPTCL-TFH
Symptom	Systemic	Systemic in ~50% of cases	NA
Morphology	Polymorphic with FDCs, HEVs, and EBV+ activated B-cells	Follicular with follicles and HRS-like cells	Poly/monomorphic
Immunology	Frequently co-expressed more than 2 Tfh markers (CXCL13, ICOS, PD-1, CD10, Bcl-6, CCR5, SAP, MAF, and CD200)		
Cytogenetics	Recurring cooccurring chromosomal gains of 5 and 21; others infrequently emerge	Frequently t(5;9)(q32;q22)	NA
Molecular biology	VAV1-STAP2, ITK-SYK, and other gene fusions	ITK-SYK (in ~40% of cases), RLTPR-FES, and ITK-FER	NA
Mutation landscape	TET2, DNMT3A, IDH2, RHOA, and TCR pathway mutations	TET2, DNMT3A, RHOA, and TCR pathway mutations	TET2, DNMT3A, RHOA, CD28, and TCR pathway mutations

AITL, angioimmunoblastic T-cell lymphoma; FTCL, follicular T-cell lymphoma; NPTCL-TFH, nodal peripheral T-cell lymphoma with T-follicular helper phenotype; FDC, follicular dendritic cell; HEV, high endothelial venule; EBV, Epstein-Barr virus; HRS, Hodgkin and Reed-Sternberg; VAV1, vav guanine nucleotide exchange factor 1; TET2, tet methylcytosine dioxygenase 2; DNMT3A, DNA methyltransferase 3 alpha; IDH2, isocitrate dehydrogenase 2; RHOA, ras homolog member A; NA, not acquired.

### 1.1 Angioimmunoblastic T-cell lymphoma (AITL)

AITL accounts for nearly 21.1% of PTCL cases, second only to PTCL-NOS. Notably, Europe (28.7%), Asia (17.9%), and North America (16%) are the areas with the highest prevalence (19). Patients with AITL consistently show systemic symptoms characterized by lymphadenopathy, hepatosplenomegaly, skin rash, immune dysregulation, and dysgammaglobulinemia (1). The expected 5-year OS of AITL is 32% due to marked chemotherapy resistance (19).

Histologically, AITL manifests a polymorphous infiltrate, typically associated with increased numbers of follicular dendritic cells (FDCs), arborizing high endothelial venules (HEVs), and scattered areas of Epstein-Barr virus (EBV)-positive activated B cells. Interestingly, EBV-positive large B cells often express EBV/LMP (latent membrane protein), CD30, and CD15 and thus resemble Hodgkin and Reed-Sternberg (HRS) cells (8).

**Figure 2:**
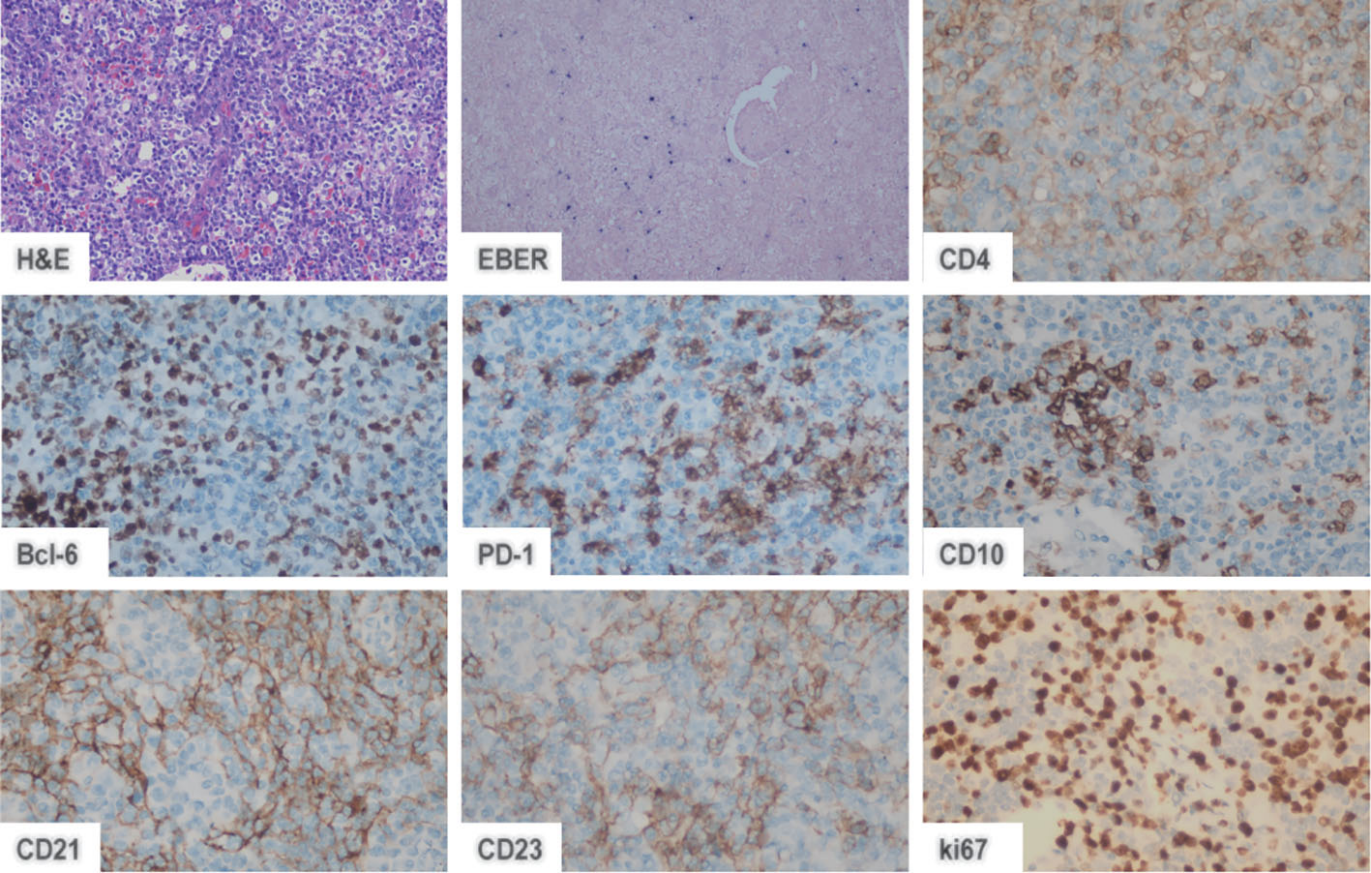
AITL immunohistochemistry patterns. In AITL, neoplastic cells are positive for CD4, CD10, Bcl-6, and PD-1, while a few scattered cells are positive for EBER.

In contrast to other PTCLs, AITL exhibits decreased genomic intricacy, with recurring cooccurring chromosomal gains of 5 and 21, whereas other aberrations (e.g., 7q, 11, 19, or 22q) occur infrequently (< 10%) (9). A study by Ibrahim et al. stated that 74% (14/19) of AITL patients coexpressed more than 2 Tfh markers. In detail, CXCL13 (89%) and ICOS (89%) were found to be more sensitive but less specific markers of AITL than PD-1 (74%), CD10 (47%), and Bcl-6 (42%) (10). Common AITL immunohistochemistry patterns are showed in **Figure 2**.

### 1.2 Follicular T-cell lymphoma (FTCL)

FTCL manifests a typical follicular growth pattern, which was considered a pattern restricted to B-cell lymphomas until 2001 (9, 11). In a study by Miyoshi et al., systemic symptoms were noted in approximately half of the patients with FTCL (12). However, hypergammaglobulinemia, skin rash, and other autoimmune manifestations are rarely found in FTCL, unlike the case in AITL (11).

FTCL mimics AITL morphologically and immunophenotypically, with HRS-like cells frequently noted. However, FTCL lacks proliferating HEVs and FDCs, with follicles occupied by abnormal T cells marked by Tfh markers (as listed above) (9).

Moreover, FTCL is genetically characterized by the t (5, 8)(q32;q22) translocation, which includes the aberrant fusion of ITK-SYK; this translocation is rarely found in AITL (9, 13). The ITK-SYK fusion protein contributes to oncogenesis through the antigen-independent phosphorylation of TCR proximal proteins (14). In addition to ITK-SYK, RLTPR-FES and ITK-FER seem exclusive to FTCL and are promising therapeutic targets (9). FTCL shares a mutation landscape with AITL, including TET2, DNMT3A, and RHOA mutations, but IDH2 mutation has not been found in FTCL (15).

### 1.3 Nodal peripheral T-cell lymphoma with T-follicular helper phenotype (NPTCL-TFH)

NPTCL-TFH, previously classified as PTCL-NOS with a Tfh immunophenotype, is a provisional category of T-FHCL (9). In contrast to AITL, NPTCL-TFH lacks the typical morphologic features of AITL, such as FDC hyperplasia and an increase in HEVs, and shows limited polymorphic or monomorphic infiltration (15). NPTCL-TFH has a similar expression and mutational profile to AITL, expressing some Tfh markers. Remarkably, similar to the case in FTCL, IDH2 mutations are rare in NPTCL-TFH (15). A T-zone pattern has been observed in several cases (20). Explicit diagnostic criteria for NPTCL-TFH need to be established.

## 2 Molecular origin of T-FHCL

T-follicular helper (Tfh) cells are acknowledged as the cellular origin of malignant transformation in T-FHCL. Over the past decade, the identification of Tfh cells has significantly improved the understanding of the cellular and molecular mechanisms of helper cells (21, 22). Tfh cells are regarded as a specific subtype of T helper (Th) cells that contribute to germinal center (GC) formation (23, 24). Tfh cells interact closely with B cells in the GC, promoting B-cell growth, affinity maturation, and immunoglobulin class switching (22, 24). Auxiliary effects are mainly achieved through cytokine and costimulatory signaling [18].

**Figure 3:**
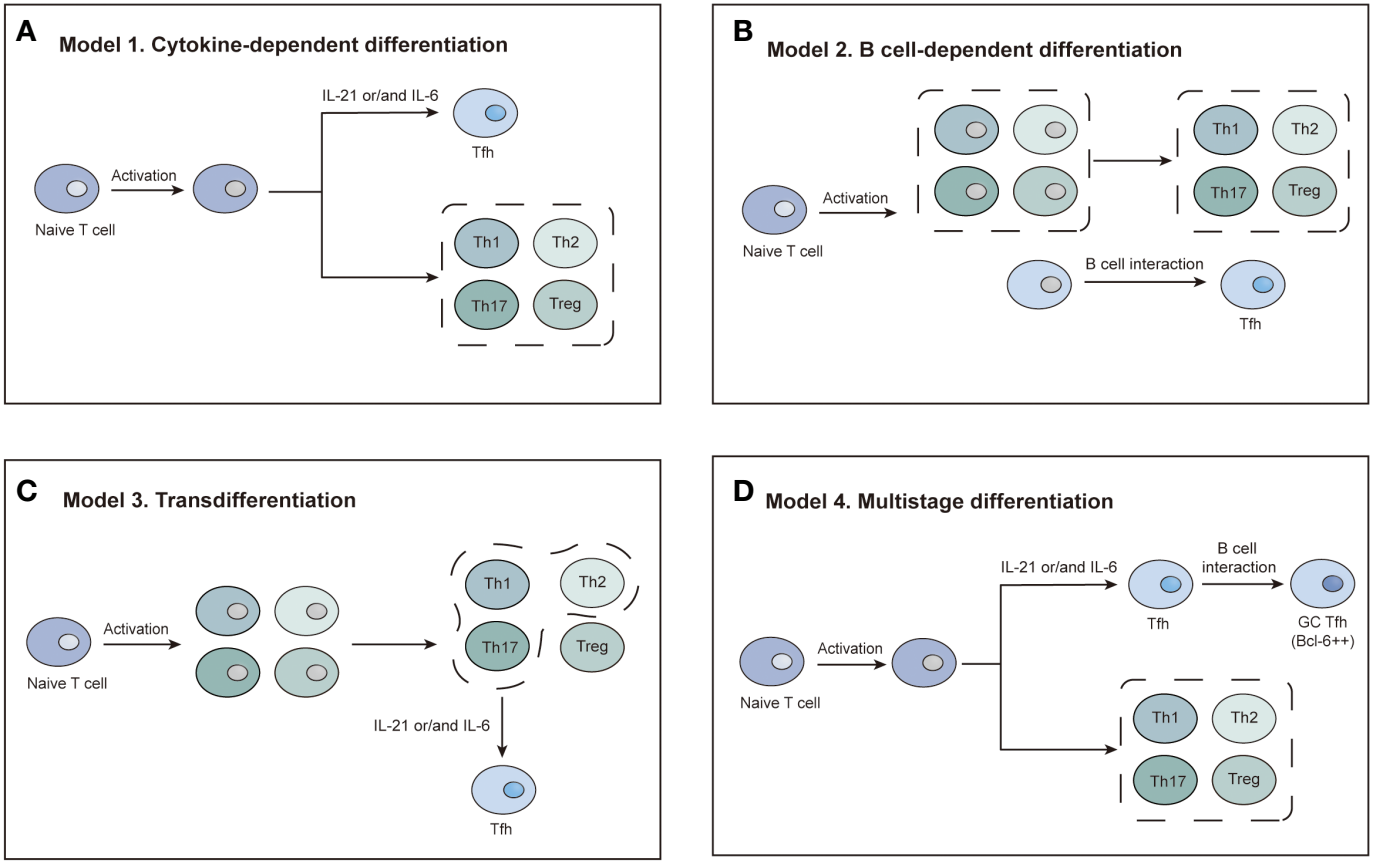
Four models of Tfh cell differentiation. **(A)** Model 1: Activated T cells differentiate into Tfh cells in the presence of IL-21 or IL-6. **(B)** Model 2: A distinct T-cell subset differentiates into Tfh cells dependent on interaction with B cells. **(C)** Model 3: Tfh differentiation is a secondary process. Activated T cells first differentiate into four subsets. The Th1, Th2, and Th17 subsets differentiate into Tfh cells *via* IL-21 or IL-6 induction. **(D)** Model 4: IL-21/6-induced signaling initiates Tfh cell differentiation. Complete polarization occurs after primary Tfh cells enter the germinal center and interact with B cells.

Tfh cell differentiation is a complicated process. Three basic differentiation models focusing on different biological properties of Tfh cells have been reported in the previous literature, based on which Crotty et al. conceived a comprehensive multistage differentiation model (**Figure 3**) (22). The first model of Tfh cell differentiation is similar to that of Th1, Th2, Th17, or Treg cells, featuring the stimulation of one or two cytokines (**Figure 3A**) (22, 25). However, this model cannot account for the normal levels of Tfh cells in IL-21- or IL-6-deficient mice and the decrease in Tfh cells in the absence of B cells (26, 27). The second model suggests that Tfh cell differentiation is B-cell-dependent (28). Tfh cells disappear after infection without B cells in a manner that is not associated with structural defects in the immune tissue caused by B-cell deficiency, demonstrating that Tfh cell differentiation is inextricably linked to the role of B cells (29). After initial antigen recognition, some CD4 T cells migrate to and interact with B cells at the T-B border, which causes T cells to express Bcl-6 and differentiate into Tfh cells (**Figure 3B**) (27, 29, 30). In the third model, Tfh cells are not considered a separate CD4 T-cell subpopulation and must undergo Th1, Th2, or Th17 differentiation (**Figure 3C**) (29–31). A comprehensive multistage differentiation model was proposed to reconcile the inconsistencies of these three models. In the fourth model, IL-21 or IL-6 induces Bcl-6 expression in Tfh cells and maintains B-cell survival to help Tfh cells differentiate (25). In the early stages of infection, Tfh cells receive antigens presented by DCs and begin initial differentiation (27, 29, 30). The interaction with DCs also promotes the expression of ICOS, which is necessary for the expression of Bcl-6 and the maintenance of the differentiated Tfh cell state (22). Bcl-6 upregulates the expression of CXCR5, which responds to CXCL13 secreted by B cells. Eventually, Tfh cells migrate to the T-B border to interact with B cells and mature (**Figure 3D**) (22, 29). The key to determining the Tfh subtypes is the emergence of Bcl-6 (21, 22). Bcl-6 is a dominant transcription factor in Tfh cell development and a master regulator of the GC reaction (21, 22, 24). Bcl-6 antagonizes the expression of Blimp-1, which promotes GC B-cell differentiation (21, 22, 24). Tfh cells will not differentiate without Bcl-6, while other CD4 T cells will not be affected (21, 22). As a result, Bcl-6 is a promising target for novel agent design (32).

Tfh cells cannot normally work without their characteristic surface markers, including CD40-CD40L, ICOS-ICOSL, and PD-1 (22, 24). The CD40-CD40L bidirectional signaling axis is significantly involved in B-cell activation, differentiation, and survival (22, 33). It provides a continuous survival signal to B cells and inhibits B-cell apoptosis (22, 33). The ICOS-ICOSL complex is vital for the formation of germinal centers and can maintain the differentiation state of Tfh cells (22). PD-1 can provide an inhibitory signal for GC Tfh cells, and high expression of PD-1 continuously stimulates B cells and inhibits B-cell apoptosis (22). Cytokines secreted by Tfh cells are also crucial. IL-21 secreted by Tfh cells is the primary promoter of B-cell proliferation and differentiation (21, 22, 24). The level of IL-21 regulates B-cell proliferation, the expression of PD-1 and CXCR5, and Tfh cell differentiation (21–24). In addition, IL-4 is vital for B-cell survival, effectively increasing B-cell glucose uptake, metabolic efficiency, and resistance to apoptosis (22).

## 3 Molecular pathogenesis of T-FHCL

The understanding of the molecular pathogenesis of T-FHCL has markedly evolved recently. However, the uncertainty about genetic heterogeneity and clonal architecture triggered by the disturbance of typical cell types could not be avoided in the sequencing of malignant lymph nodes (16). The characteristic mutations in T-FHCL mainly include the following categories: (a) epigenetic mutations, (b) mutations in ras homolog member A (RHOA), and (c) mutations in T-cell receptor (TCR) pathway genes.

### 3.1 Epigenetic mutations

Via next-generation sequencing (NGS), several recurrent epigenetic mutations have been identified in PTCL, such as mutations of TET2, DNMT3A, IDH2, MLL2, KMT2A, CREBBP, KDM6A, and EP300 (34, 35). In T-FHCL, the first three genes have the highest mutation rate and are therefore considered more important.

Tet methylcytosine dioxygenase 2 (TET2) is a tumor suppressor gene that participates in DNA demethylation and is associated with regulating the differentiation of specific Th-cell subsets (16, 36). TET2 mutations frequently occur in AITL (47%-83%), with nonsense and frameshift mutations spread over the whole TET2 protein, yet missense mutations are restricted to the C-terminal catalytic domain (17). However, these mutations are not exclusive to tumor cells and can be seen in a few nontumor cells, suggesting that the aberrations are germline mutations or that clonal hematopoiesis has occurred (16).

DNA methyltransferase 3 alpha (DNMT3A) functions in *de novo* methylation by catalyzing the transfer of methyl groups to cytosine nucleotides of CpG island DNA (34). The R882H hotspot mutation is the most common DNMT3A mutation, decreasing activity and having a dominant negative effect (17). In addition, DNMT3A mutations are frequently accompanied by TET2 mutations (in 70%~100% of AITL cases), showing synergistic effects on lymphomagenesis despite having opposite epigenetic effects (14).

Isocitrate dehydrogenase 2 (IDH2) catalyzes the conversion of isocitric acid into α-ketoglutaric acid (α-KG). Nevertheless, IDH2 mutants aberrantly promote the production of D-2-hydroxyglutarate (D-2-HG) in place of α-KG. D-2-HG interferes with a subset of α-KG-dependent dioxygenases, including Jumonji-C histone demethylases (17). IDH2 mutations occur in 20%-45% of AITL cases, most affecting the 172^nd^ residue (arginine; R172), which appears to be exclusive to AITL (14, 16, 17). In a T-cell line, IDH2 R172 mutations result in the hypermethylation of gene promoters (16). Similar to DNMT3A mutations, IDH2 mutations also frequently cooccur with TET2 mutations (in 70%-90% of cases) (14).

### 3.2 Mutations in RHOA

RHOA, a small guanine nucleotide triphosphate (GTP)-binding protein, functions as a molecular switch and is regulated by GTPase-activating proteins (GAPs), guanine nucleotide exchange factors (GEFs), and guanine nucleotide dissociation inhibitors (GDIs) (16). RHOA is involved in various biological processes, including actin polymerization, cytoskeleton remodeling, adhesion, cytokinesis, proliferation, and cell death (14, 16, 17, 37).

Seemingly restricted to tumor cells (38), RHOA mutations are present in approximately 50-70% of AITL cases, with the G17V missense mutation (valine substitution for the 17^th^ residue (glycine)), accounting for 91% of RHOA mutations (14). The mutant protein encoded by RHOA G17V mutation lacks GTP binding capacity and shows a dominant negative effect, reducing the activity of wild-type RHOA (16). It has been reported that RHOA G17V mutations lead to the sequestration and obstruction of GEFs in classical RHOA signaling, which hinders GTP binding (14). Recently, another essential oncogenic mechanism of RHOA G17V mutations has been revealed: the G17V mutant directly binds to VAV1, a critical intermediate of the TCR pathway, and then aberrantly activates the TCR pathway, and this finding offers insights into AITL development (17, 18). Moreover, in a murine study, RHOA G17V mutations were found to induce Tfh lineage specification and AITL transformation of CD4+ T cells by promoting the overexpression of ICOS and Bcl-6 and activating the PI3K and MAPK pathways (14, 37).

RHOA G17V mutations often cooccur with at least one epigenetic mutation (TET2, DNMT3A, or IDH2 mutation) in up to 94% of cases (14). A meta-analysis of RHOA mutations in AITL showed that RHOA-mutated cases had a considerably higher likelihood of carrying IDH2 and TET2 mutations than RHOA-wild-type cases, but DNMT3A mutations lacked a similarly strong correlation with RHOA mutations (38). A synergistic effect between TET2 and RHOA G17V in inducing tumor cell proliferation was discovered in mouse models of AITL with TET2 deletion and RHOA G17V mutation (14, 37).

In addition to RHOA G17V, other RHOA mutations detected in AITL cases mainly include p.T19I (39), p.K18N (>3%), p.S26R (~1%), and p.C20W (~1%) (14). Interestingly, the p.K18N mutant has a higher capacity for GTP binding, which means that the means by which classical RHOA signaling is disrupted to trigger AITL remains to be revealed (17). RHOA mutations are not specific to AITL and also occur in diffuse gastric carcinoma (p.Y42C and p.R5Q), Burkitt lymphoma (p.Y42C and p.R5Q), and ATLL (p.C16R) (17).

### 3.3 Mutations in TCR pathway genes

The T-cell receptor (TCR) signaling pathway is essential for T-cell development, activation, and immunological tolerance (40). It is aided by several effector enzymes and adaptor proteins, which can be easily mutated and cause TCR signaling dysregulation (40). In addition to resulting in anergy or autoimmunity, TCR signaling dysregulation can influence and induce T-FHCL development (40). Most of these mutations appear to be activating and mutually exclusive (16).

In healthy humans, natural TCR polyclonally occurs due to the stimulation of endogenous and exogenous antigens. In a population-based immunohistochemical study, the clonal TCR-γ rearrangements observed in AITL cases (n=32) were typically monoclonal (56%), while FTCL cases (n=4) showed either polyclonal (50%) or clonal with background (50%) (41). The aberrant outcomes associated with TCR rearrangements may provide insights into the pathogenesis of T-FHCL.

CD28, as a critical costimulatory receptor for TCR-mediated activation, promotes the production of cytokines and the growth of T cells in response to ligand binding and TCR stimulation (14). Mutations in the CD28 gene, which are present in 4%-12% of AITL cases, contribute to the hyperactivation and amplification of CD28 signaling *via* enhanced affinity for intracellular adaptor proteins (with the p.T195P/I mutation) or the CD28 ligands CD80 and CD86 (with the p.D124E/V mutation) (14, 17). Both CD28 mutants affect NF-κB activation and augment signal transduction (14). The p.D124E/V CD28 mutants have a higher affinity for ICOS binding, resulting in the formation of ICOS-CD28 fusion proteins. In addition, CD28-CTLA4 fusion proteins can be detected in 38% of AITL cases and show stimulatory rather than inhibitory signals for T-cell activation (14).

FYN, a protein tyrosine kinase (PTK) in the TCR pathway, plays a pivotal role alongside LCK in the activation of TCR signaling through the tyrosine phosphorylation of CD3 (40). FYN mutations are found in 2.8%-4% of AITL cases and can increase PTK signaling and induce AITL oncogenesis (14). Moreover, the FYN-TRAF3IP2 gene fusion was identified in AITL; it upregulates canonical NF-κB signaling upon TCR activation and induces NF-κB-driven T-cell transformation in murine hematopoietic progenitor models (14, 42).

The phospholipase C gamma 1 (PLCγ1) promotes Ca2+-dependent calcineurin NFAT (nuclear factor of activated T cells) pathway activity (14, 40, 43). In AITL, PLCγ1 mutations are the most common TCR pathway mutation, found in 11.1%-14.1% of cases (14). PLCγ1 mutations have been shown to promote MALT1 cleavage, hyperactivate NFAT signaling, and enhance TCR signaling (14, 43).

Vav guanine nucleotide exchange factor 1 (VAV1) serves as a GEF-independent adaptor to enhance the phosphorylation of PLCγ1 (14, 43). Furthermore, VAV1 functions as a GEF for RHOA, CDC42, and RAC1 (14) after a conformational change provoked by specific phosphorylation at Tyr160 and Tyr142 (14, 43). In AITL, VAV1 mutations are found in 4.7%-5.6% of cases, with p.D797G and p.Y826S mutations affecting the C-terminal SH3 domain (14). RHOA G17V can induce VAV1 Tyr174 hyperphosphorylation and facilitate binding with VAV1 regardless of TCR stimulation, but RHOA G17V seldom coexists with VAV1 mutation (14, 18). In addition, the VAV1-STAP2 gene fusion protein, which is found in 8.2% of AITL patients without RHOA mutation, also induces Tyr174 hyperphosphorylation and promotes PLCγ1 phosphorylation even in the absence of TCR stimulation (14, 18). Notably, the RAC1 inhibitor azathioprine appears to target VAV1 fusion (43).

## 4 Targeted therapy for T-FHCL

**Figure 4:**
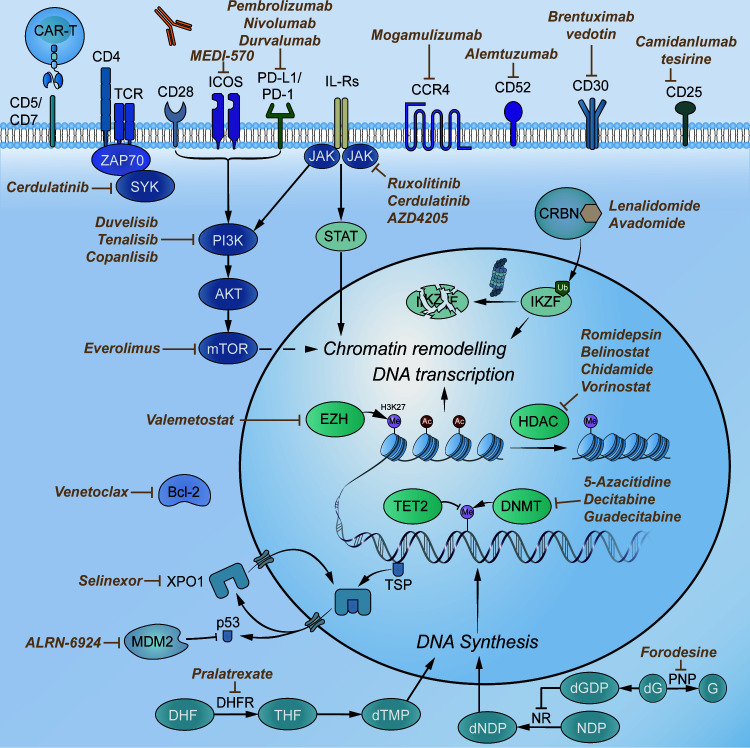
Selected novel agents for T-FHCL. Abbreviations: CD, cluster of differentiation; TCR, T-cell receptor; CCR4, chemokine receptor 4; PD-1, programmed death receptor 1; PD-L1, programmed cell death-ligand 1; PI3K, phosphoinositide 3-kinase; JAK, Janus kinase; SYK, spleen tyrosine kinase; mTOR, mammalian target of rapamycin; STAT, signal transducer and activator of transcription; CRBN, cereblon; IKZF, Ikaros zinc finger; HDAC, histone deacetylase; DNMT, DNA methyltransferase; EZH, enhancer of zeste homolog; DHFR, dihydrofolate reductase; NR, ribonucleotide reductase; G, guanine; dG, deoxyguanosine; dNDP, deoxyribonucleoside diphosphate; PNP, purine nucleoside phosphorylase; TSP, tumor suppression protein; MDM2, murine double minute 2; XPO1, exportin 1.

The transmission of various biological signals requires multiple biomarkers, and these biomarkers can be targeted by novel therapies to improve the prognosis of T-FHCL (**Figure 4**). We searched Medline and Embase for controlled trials and systematic reviews of novel agents for T-FHCL up to July 13, 2022. In total, 69 clinical trials or retrospective studies of targeted therapies for T-FHCL were assessed. The overall response rate (ORR) and PFS were assessed. The characteristics of the clinical and preclinical trials for different categories of T-FHCL agents are illustrated in **Tables 2**–**6**. Further details can be found on ClinicalTrials.gov.

**Table 2:** Novel and experimental agents targeting surface molecules in T-FHCL.

Year	Agent	Drug target	Signaling pathway	Phase	Disease	ORR	mPFS (months)	Clinical trial registration number	Reference
2020	MEDI-570	ICOS	mTOR pathway	I	R/R AITL	33% (4/12)	NA	NCT02520791	(44)
2019	Pembrolizumab	PD-1	mTOR, Ras/EMK/ERK pathway	II	R/R MTCL (T-FHCL, n=4)	33% (5/15)	3.2	NCT02535247	(45)
2020	Pembrolizumab + romidepsin	PD-1, HDAC	mTOR, Ras/EMK/ERK pathway	II	R/R PTCL (T-FHCL, n=5), MF (n=3)	50% (10/20)	NA	NCT03278782	(46)
2017*	Durvalumab + pralatrexate/romidepsin/5-azacitidine	PD-L1	mTOR, Ras/EMK/ERK pathway	I/IIa	R/R PTCL	NA	NA	NCT03161223	NA
2019	Nivolumab	PD-L1	mTOR, Ras/EMK/ERK pathway	II	R/R PTCL (AITL, n=6)	33% (4/12)	1.9	NCT03075553	(47)
2014	Brentuximab vedotin	CD30	MAPK/NF-κB pathway	II	Relapsed AITL	54% (7/13)	6.7	NCT01421667	(48)
2014	Brentuximab vedotin + CHP/CHOP	CD30	MAPK/NF-κB pathway	I	Untreated CD30+ AITL	100% (2/2)	10.9	NCT01309789	(49)
2021	Alemtuzumab + CHOP	CD52	TCR pathway	III	Untreated PTCL (AITL, n=24)	72% (42/58)	5-year PFS 23%	NCT00725231	(50)
2014	Mogamulizumab	CCR4	ERK/NF-κB/MMP13 pathway	II	Relapsed PTCL (AITL, n=12)	34% (10/29)	2.0	NCT01192984	(51)
2016				II	R/R PTCL (AITL, n=12)	11.4% (4/35)	2.1	NCT01611142	(52)
2021	Camidanlumab tesirine	CD25	IL-2-IL2R pathways	I	R/R T-NHL (AITL, n=3)	48% (15/31)	2.7	NCT02432235	(53)

*The first posted year of the clinical trial.

NA, not acquired; ORR, overall response rate; mPFS, median progression-free survival; T-FHCL, T-follicular helper cell lymphoma; AITL, angioimmunoblastic T-cell lymphoma; PTCL, peripheral T-cell lymphoma; R/R, relapsed/refractory; MTCL, mature T-cell lymphoma; T-NHL, T-cell non-Hodgkin lymphoma; MF, mycosis fungoides; CD, cluster of differentiation; CCR4, chemokine receptor 4; ICOS, inducible T-cell costimulator; mTOR, mammalian target of rapamycin; PD-1, programmed death receptor 1; EMK, ELKL motif kinase; ERK, extracellular regulated protein kinase; NF-κB, nuclear factor kappa-B; MAPK, mitogen-activated protein kinase; CHOP, cyclophosphamide, doxorubicin, vincristine, and prednisone; CHP, cyclophosphamide, doxorubicin, and prednisone.

**Table 3:** Novel and experimental agents targeting kinases in T-FHCL.

Year	Agent	Drug target	Signaling pathway	Phase	Disease	ORR	mPFS (months)	Clinical trial registration number	Reference
2018	Duvelisib	PI3K δ/γ	mTOR pathway	I	R/R PTCL (AITL, n=3)	50% (8/16)	8.3	NCT01476657	(54)
2020				II	R/R PTCL	40% (8/20)	NA	NCT03372057	(55)
2017	Duvelisib + romidepsin/bortezomib	PI3K δ/γ, HDAC	mTOR pathway	I	R/R TCL	50% (4/8)/53% (8/15)	NA	NCT02783625	(56)
2020	Tenalisib	PI3K δ/γ	mTOR pathway	I/Ib	R/R PTCL	46.7% (7/15)	NA	NCT02567656	(57)
2017	Copanlisib	PI3K α/δ	mTOR pathway	II	R/R PTCL	21.4% (3/14)	NA	NCT01660451	(58)
2020	Copanlisib + gemcitabine	PI3K α/δ	mTOR pathway	I/II	R/R AITL	77.8% (7/9)	13.0	NCT03052933	(59)
2021*	BEBT-908	PI3K, HDAC	mTOR pathway	II	R/R PTCL	NA	NA	ChiCTR-TNC-20210170	NA
2015	Everolimus	mTOR	mTOR pathway	II	Relapsed TCL (AITL, n=1)	44% (7/16)	4.1	NCT00436618	(60)
2016	Everolimus + CHOP	mTOR	mTOR pathway	II	Untreated PTCL (AITL, n = 3)	90% (27/30)	11	NCT01198665	(61)
2021	Ruxolitinib	JAK1/2	JAK/STAT pathway	II	R/R T-FHCL	33% (3/9)	NA	NCT02974647	(62)
2020	Golidocitinib	JAK1	JAK/STAT pathway	I/II	R/R PTCL (AITL, n=10)	40.9% (9/22)	NA	NCT04105010	(63)
2019	Cerdulatinib	JAK, SYK	JAK/STAT pathway	II	R/R T-FHCL	55% (12/22)	NA	NCT04021082	(64)
2021				IIa	R/R PTCL (T-FHCL, n=27)	36.2% (21/58)	NA	NCT01994382	(65)
2015	Alisertib	AAK	AKT/mTOR/AMPK/p38 pathway	II	R/R PTCL (AITL, n=9)	30% (9/30)	NA	NCT01466881	(66)
2019				III	R/R PTCL (AITL, n=31)	33% (34/102)	3.8	NCT01482962	(67)
2020	Dasatinib	LYN, FYN	TCR pathway	I	R/R AITL	80% (4/5)	NA	UMIN000025856	(68)

*The first posted year of the clinical trial.

NA, not acquired; ORR, overall response rate; mPFS, median progression-free survival; T-FHCL, T-follicular helper cell lymphoma; AITL, angioimmunoblastic T-cell lymphoma; PTCL, peripheral T-cell lymphoma; TCL, T-cell lymphoma; R/R, relapsed/refractory; PI3K, phosphoinositide 3-kinase; AAK, aurora A kinase; JAK, Janus kinase; SYK, spleen tyrosine kinase; mTOR, mammalian target of rapamycin; AMPK, adenosine monophosphate-activated protein kinase; HDAC, histone deacetylase; STAT, signal transducer and activator of transcription; TCR, T-cell receptor; CHOP, cyclophosphamide, doxorubicin, vincristine, and prednisone.

**Table 4:** Novel and experimental agents targeting epigenetic enzymes in T-FHCL.

Year	Agent	Drug target	Phase	Disease	ORR	mPFS (months)	Clinical trial registration number	Reference
2017	Romidepsin	HDAC	II	R/R AITL	44% (8/18)	5.6	NCT01456039	(69)
2022	Romidepsin + CHOP	HDAC	III	Untreated PTCL (AITL, n=101)	63% (133/211)	12.0	NCT01796002	(70)
2015	Belinostat	HDAC	II	R/R AITL	45.5% (10/22)	1.6	NCT00865969	(71)
2021	Belinostat + CHOP	HDAC	I	Untreated AITL	89% (9/10)	NA	NCT01839097	(72)
2015	Chidamide	HDAC-I/II	II	R/R AITL	50% (5/10)	NA	ChiCTR-TNC-10000811	(73)
2021	Chidamide + CHOP	HDAC-I/II	I	Untreated PTCL (T-FHCL, n=9)	89.3% (25/28)	14.0	NCT02809573	(74)
2022	Chidamide + prednisone + etoposide + thalidomide	HDAC-I/II	II	Untreated AITL	90.2% (46/51)	42.6	NCT03273452	(75)
2021*	Chidamide + 5-azacitidine + CHOP	HDAC-I/II, DNMT	III	Untreated PTCL	NA	NA	NCT05075460	NA
2013	Vorinostat + CHOP	HDAC	II	Untreated PTCL (AITL, n=5)	85.7% (12/14)	31	NCT00787527	(76)
2018	5-Azacitidine	DNMT	II	R/R AITL	75% (9/12)	15	EudraCT2017-003909-17	(77)
2021	5-Azacitidine + romidepsin	DNMT, HDAC	II	Untreated or R/R T-FHCL	80% (12/15)	8.9	NCT01998035	(78)
2022	Guadecitabine	DNMT	II	Untreated or R/R T-FHCL (AITL, n=11)	43.8% (7/16)	2.9	ACTRN12618000028202	(79)
2017*	Decitabine + pembrolizumab + pralatrexate	DNMT	Ib	R/R PTCL, CTCL	NA	NA	NCT03240211	NA
2018*	Decitabine + CHOP	DNMT	III	Untreated PTCL	NA	NA	NCT03553537	NA
2017	Valemetostat	EZH1/2	I	R/R TCL (AITL, n=2)	80% (4/5)	NA	NCT02732275	(80)
2014*	Enasidenib	IDH2	I/II	R/R AITL (n=5)	NA	NA	NCT02273739	NA

*The first posted year of the clinical trial.

NA, not acquired; ORR, overall response rate; mPFS, median progression-free survival; T-FHCL, T-follicular helper cell lymphoma; AITL, angioimmunoblastic T-cell lymphoma; PTCL, peripheral T-cell lymphoma; CTCL, cutaneous T-cell lymphoma; R/R, relapsed/refractory; HDAC, histone deacetylase; DNMT, DNA methyltransferase; EZH, enhancer of zeste homolog; IDH2, isocitrate dehydrogenase 2; CHOP, cyclophosphamide, doxorubicin, vincristine, and prednisone.

**Table 5:** Novel and experimental CAR immunotherapies for T-cell lymphoma.

Year	Agent	Drug target	Signaling pathway	Phase	Disease	Therapeutic outcomes	Clinical trial registration number	Reference
2021*	CD4 CAR-T cells	CD4	TCR pathway	I	CD4+ R/R PTCL	NA	NCT04712864	NA
2019	CD5 CAR-T cells	CD5	TCR pathway	I	CD5+ T-ALL (n=4), CD5+ T-NHL (n=5)	ORR 44.4% (4/9) and CRR 33.3% (3/9)	NCT03081910	(81)
2021*	CT125A (Fully Human CD5 CAR-T cells)	CD5	TCR pathway	Early I	CD5+ R/R TCL	NA	NCT04767308	NA
2021*	MT-101 (CD5 ATAK cells)	CD5	TCR pathway	I/II	CD5+ R/R PTCL	NA	NCT05138458	NA
2022	CD7 CAR-T cells	CD7	PKC/PTK/mTOR pathway	I	R/R T-ALL (n=14), R/R T-LBL (n=6)	Intramedullary CR 95.0% (19/20) and extramedullary CR 55.6% (5/9)	NCT04572308	(82)
2020*				I	CD7+ R/R PTCL	NA	NCT04480788	NA
2016*	CD7 CAR-pNK cells	CD7	PKC/PTK/mTOR pathway	I/II	CD7+ R/R PTCL	NA	NCT02742727	NA
2019*	CD30 CAR-T cells	CD30	MAPK/NF-κB pathway	II	CD30+ R/R PTCL	NA	NCT04083495	NA
2019*				I	CD30+ R/R PTCL	NA	NCT04008394	NA
2020*				I	CD30+ R/R PTCL	NA	NCT04526834	NA
2022*				I	CD30+ R/R PTCL	NA	NCT05208853	NA
2017*	Fully human CD30 CAR-T cells	CD30	MAPK/NF-κB pathway	I	CD30+ advanced PTCL	NA	NCT03049449	NA
2018*	AUTO4 (RQR8/aTRBC1 CAR-T cells)	TRBC1	TCR pathway	I	TRBC1+ R/R T-NHL	NA	NCT03590574	NA
2021*	TRBC1 CAR-T cells	TRBC1	TCR pathway	I	TRBC1+ R/R PTCL	NA	NCT04828174	NA

*The first posted year of the clinical trial.

CAR-T, chimeric antigen receptor T; CAR-pNK, chimeric antigen receptor pNK; NA, not acquired; ORR, overall response rate; CRR, complete response rate; R/R, relapsed/refractory; PTCL, peripheral T-cell lymphoma; T-ALL, T-acute lymphoblastic leukemia; T-NHL, T-non-Hodgkin lymphoma; TCL, T-cell lymphoma; T-LBL, T-cell lymphoblastic lymphoma; mTOR, mammalian target of rapamycin; TCR, T-cell receptor; MAPK, mitogen-activated protein kinase; PKC, protein kinase C; PTK, protein tyrosine kinase; NF-κB, nuclear factor kappa-B; CD, cluster of differentiation; TRBC1, T-cell receptor β-chain constant domain 1.

**Table 6:** Other novel targeted agents in T-FHCL.

Year	Agent	Drug target	Signaling pathway	Phase	Disease	ORR	mPFS (months)	Clinical trial registration number	Reference
2021	Lenalidomide + CHOP	CRBN	CRL4-IKZF pathway	II	Untreated PTCL (T-FHCL, n=71; AITL, n=67), DLBCL (n=1)	56% (44/78)	14.1	NCT01553786	(83)
2018	Lenalidomide + CHOEP	CRBN	CRL4-IKZF pathway	II	Untreated PTCL (AITL, n=12)	86.7% (26/30)	1-year PFS 68%	NCT02561273	(84)
2014	Lenalidomide + vorinostat + dexamethasone	CRBN, HDAC	CRL4-IKZF pathway	I/II	R/R PTCL (AITL, n=5)	25% (2/8)	2.2	NCT00972842	(85)
2021	Avadomide	CRBN	CRL4-IKZF pathway	I	Advanced NHL (AITL, n=1)	54% (7/13)	46.1	NCT01421524	(86)
2017	Pralatrexate	DHFR	Folate pathway	I/II	R/R AITL	44.4% (4/9)	5.6	NCT02013362	(87)
2019				III	R/R AITL	55% (11/20)	4.8	NCT03349333	(88)
2019	Forodesine	PNP	Nucleotide salvage pathway	I/II	R/R AITL	33% (6/18)	NA	NCT01776411	(89)
2019	Tipifarnib	FTase	CXCL12/CXCR4 pathway	II	R/R AITL	45.5% (5/11)	NA	NCT02464228	(90)
2021	Venetoclax	Bcl-2	p53 pathway	II	R/R PTCL (AITL, n=4)	18% (3/17)	3.8	NCT03552692	(91)
2017	Selinexor	XPO1	p53/RB1/p27 pathway	I	R/R PTCL	50% (1/2)	NA	NCT01607892	(92)
2020	Selinexor + DICE	XPO1	p53/RB1/p27 pathway	I	R/R TCL (T-FHCL, n=6; AITL, n=5), NKTL (n=1)	100% (10/10)	NA	NCT03212937	(93)
2021	ALRN-6924	MDM2, MDMX	p53 pathway	I	Advanced PTCL with wild-type TP53	100% (1/1)	NA	NCT02264613	(94)

NA, not acquired; ORR, overall response rate; mPFS, median progression-free survival; T-FHCL, T-follicular helper cell lymphoma; AITL, angioimmunoblastic T-cell lymphoma; PTCL, peripheral T-cell lymphoma; NHL, non-Hodgkin lymphoma; TCL, T-cell lymphoma; DLBCL, diffuse large B-cell lymphoma; NKTL, natural killer/T-cell lymphoma; R/R, relapsed/refractory; CRBN, cereblon; HDAC, histone deacetylase; DHFR, dihydrofolate reductase; PNP, purine nucleoside phosphorylase; FTase, farnesyltransferase; MDM2, murine double minute 2; MDMX, murine double minute X; Bcl-2, B-cell lymphoma-2; XPO1, exportin 1; CRL4, Cullin4-RING ligase; IKZF, Ikaros zinc finger; CXCL12, C-X-C motif chemokine ligand 12; CXCR4, C-X-C motif chemokine receptor 4; RB1, RB transcriptional corepressor 1; CHOP, cyclophosphamide, doxorubicin, vincristine, and prednisone; CHP, cyclophosphamide, doxorubicin, and prednisone; CHOEP, cyclophosphamide, doxorubicin, vincristine, etoposide, and prednisone; DICE, dexamethasone, ifosfamide, carboplatin, and etoposide.

### 4.1 Surface molecule antibodies

Neoplastic surface antigens are usually associated with the molecular origin of tumor cells. T-FHCL is no exception and has high expression of ICOS and PD-1 and varying expression levels of other surface molecules. MEDI-570 is a human antagonistic fucosylated IgG1 kappa monoclonal antibody directed against ICOS. In a phase I study of MEDI-570 (NCT02520791), 4 of 12 relapsed/refractory (R/R) AITL patients achieved partial remission (PR), and 7 achieved stable disease (SD), with anemia (12%) and hypophosphatemia (12%) recorded as the most common adverse events (AEs) (44). Despite promising clinical activity, the safety and efficacy of MEDI-570 need to be further researched in the expansion phase.

The PD-1/PD-L1 pathway, which functions as an immune checkpoint, has recently been a focus of cancer therapy research. The anti-PD-1 antibody pembrolizumab (MK-3475) showed modest single-agent activity in R/R mature T-cell lymphoma, achieving a 33% (5/15) ORR with 4 complete responses (CRs) and 1 partial response (PR) and a median PFS of 3.2 months. The most common grade 3 treatment-emergent AEs were rash and pneumonitis (11%; 2 each) (45). It was reported that neither PD-L1 nor p-AKT was linearly associated with outcomes (45). However, another study of pembrolizumab in combination with romidepsin (NCT03278782) found the opposite: the patients who achieved CR had a higher level of PD-L1 than those who achieved PR or SD (p=0.048) (46). In a phase II study (NCT03075553), the anti-PD-L1 antibody nivolumab showed an unremarkable ORR of 16.7% (1/6) in R/R AITL (47). This study was halted due to moderate activity and a short duration of response (DoR) (47).

In addition to the antibodies targeting ICOS and PD-1/PD-L1, various agents targeting other surface molecules have been investigated in the treatment of T-FHCL. Brentuximab vedotin (BV) is a conjugate containing an anti-CD30 monoclonal antibody and a microtubule-disrupting agent, monomethyl auristatin E (MMAE). A 54% (7/13) ORR (including 5 CRs and 2 PRs) and mPFS of 6.7 months were achieved for patients with relapsed AITL treated with single-agent BV, with grade 3 adverse events of neutropenia (14%), peripheral sensory neuropathy (9%), and hyperkalemia (9%) (48). In a prospective, randomized phase III study (NCT00725231), alemtuzumab (CAMPATH-1H), a human-mouse chimeric anti-CD52 antibody, was added to the CHOP regimen (A-CHOP), which had increased ORR (72% *vs.* 66%), 5-year PFS (22% *vs.* 13%) and confirmed/unconfirmed complete response (CR/CRu) rate (60% *vs.* 43%) compared to the CHOP regimen alone in untreated PTCL (50). However, the results showed no significant differences (50). The anti-CCR4 antibody mogamulizumab (KW-0761) achieved a 34% (10/29) ORR in relapsed PTCL along with a higher ORR (50%, 6/12) in relapsed AITL (51). But in another phase II trial (NCT01611142), mogamulizumab lacked adequate efficacy with an 11.4% (4/35) ORR in R/R PTCL and a 16.7% (2/12) ORR in R/R AITL (52). The mPFS was similar in both trials (2.0 months vs. 2.1 months) (51, 52). The anti-CD25 antibody camidanlumab tesirine (ADCT-301) exhibited clinical benefits in R/R T-cell NHL with a 48% (15/31) ORR and a 2.7-month mPFS (53). However, due to a lack of clinical evidence, the efficacy of camidanlumab tesirine for T-FHCL has not been determined (53).

### 4.2 Kinase inhibitors

Kinases in cancer-associated pathways are common targets of therapy. In T-FHCL entities, the PI3K/AKT/mTOR pathway and JAK/STAT pathway are attached much attention due to their activation and cooperativity.

The PI3K/AKT/mTOR pathway regulates many biological processes, including growth, proliferation, cell cycle progression, motility, adhesion, and angiogenesis (95). Duvelisib (IPI-145), an oral dual inhibitor of phosphoinositide 3-kinase-δ/γ (PI3K-δ/γ), showed a 50% (8/16) ORR in R/R PTCL, with 2 patients with AITL achieving CR and PR in a phase I trial (NCT01476657) (54). Notably, duvelisib plus romidepsin or bortezomib was evaluated in R/R TCL (NCT02783625): there was a 50% (4/8) ORR in the romidepsin arm and a 53% (8/15) ORR and a 20% (3/15) CR rate (CRR) in the bortezomib arm (56). Another novel oral dual PI3K-δ/γ inhibitor, tenalisib (RP6530), achieved a 46.7% (7/15) ORR in R/R PTCL, preliminarily showing a lower incidence of AEs such as neutropenia and transaminitis than duvelisib (57). The PI3K-α/δ inhibitor copanlisib achieved a 21.4% (3/14) ORR, 2 CRs and 1 PR in R/R PTCL, but the addition of gemcitabine might lead to a higher CRR and longer PFS, especially in R/R AITL (58, 59). In addition, the dual inhibitor BEBT-908, which targets PI3K and HDAC, is a promising candidate for T-FHCL. Mammalian target of rapamycin (mTOR) functions as a signal transduction center and is inhibited by everolimus (RAD001) (61). In a phase II study (NCT01198665), everolimus was added to the CHOP regimen, achieving a 90% (27/30) ORR in untreated PTCL and a 100% (3/3) CRR in untreated AITL (61).

The JAK/STAT pathway mediates signal transduction by cytokines, growth factors, and hormones (96). The JAK1/2 inhibitor ruxolitinib demonstrated a 33% (3/9) ORR in R/R T-FHCL, with 1 CR and 2 PRs (62). The highly selective JAK1 inhibitor golidocitinib (DZD4205, AZD4205) showed a 40.9% (9/22) ORR in R/R PTCL with common AEs of thrombocytopenia and neutropenia (63). The dual SYK/JAK inhibitor cerdulatinib (ALXN2075) was reported to achieve a 55% (12/22) ORR and a 41% (9/22) CRR in R/R T-FHCL (64). In another phase IIa trail (NCT01994382), cerdulatinib as monotherapy resulted in a superior prognosis in the T-FHCL subgroup (n=27) compared to the overall R/R PTCL cohort (n=58) in terms of ORR (51.9% vs. 36.2%) and CRR (37.0% vs. 20.7%) (65). The mPFS for the T-FHCL subgroup was estimated to be 4.6 months (65).

In addition, alisertib (MLN8237) inhibits aurora A kinase (AAK), which is essential for mitosis, and was evaluated in a randomized phase III study (NCT01482962); treatment resulted in a 33% (34/102) ORR in R/R PTCL (28% ORR for AITL) and an mPFS of 115 days (67). Moreover, dasatinib, which targets LYN and FYN in the TCR pathway, was initially reported to improve the survival of AITL model mice and achieved an 80% (4/5) ORR in patients with R/R AITL (68).

### 4.3 Epigenetic inhibitors

Epigenetic dysregulation plays a pivotal role in the oncogenesis of T-FHCL. Therefore, novel drugs targeting epigenetic mediators have been the focus of research, especially HDACis and DNMTis.

HDACis drive histone or non-histone protein acetylation, which promotes the generation of an open state of chromatin that facilitates gene expression (e.g., the endogenous inhibitor of cell cycle progression p21), activates transcriptional activators (e.g., p53), and suppresses transcriptional repressors (e.g., Bcl-6) (97, 98). In addition, HDACis exert antitumor efficiency by relaxing DNA and repressing gene transcription, disrupting chaperone protein function, generating free radicals, and inducing DNA damage (98). The ORRs for romidepsin, belinostat (PXD-101), and chidamide (tucidinostat, HBI-8000, CS055) as single agents ranged between 44% and 50% in the management of R/R AITL (69, 71, 73). Notably, in a phase III study of untreated PTCL patients (NCT01796002), the addition of romidepsin to CHOP (Ro-CHOP) did not improve PFS (12.0 months vs. 10.2 months, Ro-CHOP vs. CHOP), ORR (63% vs. 60%), or OS (51.8 months vs. 42.9 months) but resulted in more AEs (70). Nevertheless, belinostat plus CHOP or chidamide plus CHOP both achieved an 89% (9/10 or 25/28) ORR in untreated AITL or PTCL patients, respectively (72, 74). Of note, chidamide plus prednisone, etoposide, and thalidomide (CPET regimen) exhibited marked therapeutic outcomes in untreated AITL, with a 90.2% (46/51) ORR, a 54.9% (28/51) CRR, and a 42.6-month mPFS (75). Neutropenia (32.3%) was reported as the most common grade 3/4 AE (75). The efficacy of vorinostat plus CHOP in untreated PTCL was evaluated in 2013, showing an 85.7% (12/14) ORR and a 31-month mPFS (76). However, subsequent research mainly focused on vorinostat in the treatment of CTCL (76). Hematological and gastrointestinal toxicities are the most common AEs reported in treatment with HDACis (97).

DNMTis, which are hypomethylating agents, hinder the DNA methylation of CpG sequences to maintain gene expression (97). 5-Azacitidine (CC-486) achieved a 75% (9/12) ORR and a 15-month mPFS in R/R AITL, but 50% (6/12) of patients received additional rituximab due to the presence of active EBV replication or numerous EBV-positive B-blasts in the lymph node biopsy (77). In a multicenter phase II study (NCT01998035), combined 5-azacytidine and romidepsin achieved high ORR (61%, 14/23) and CRR (48%, 11/23) in untreated and R/R PTCL, notably with an 80% (12/15) ORR and a 60% (9/15) CRR in T-FHCL (78). Patients with T-FHCL showed a longer mPFS (8.9 months) than those with other PTCL subtypes (2.3 months) (78). *Via* next-generation sequencing, mutations of genes involved in DNA methylation, histone methylation, or histone acetylation were found more frequently in patients responding to 5-azacytidine plus romidepsin (78). Guadecitabine (SGI-110), an oligonucleotide decitabine prodrug, is superior to decitabine in terms of *in vivo* DNA demethylation. Guadecitabine showed a 43.8% (7/16) ORR and a 2.9-month mPFS in untreated and R/R T-FHCL, but there was no significant difference in ORR and mPFS between PTCLs of Tfh origin and other histologic origins (79). RHOA G17V mutations appear to be associated with improved mPFS for guadecitabine in PTCL (79). Decitabine has been utilized in combination therapy for PTCL in two trials (NCT03240211 and NCT03553537), but the results are unavailable.

EZH (enhancer of zeste homolog) and IDH are also possible targets. EZH induces trimethylation of Lys27 of histone H3 (H3K27), and valemetostat (DS-3201b, an EZH inhibitor) showed an 80% (4/5) ORR in R/R TCL (AITL, n=2) (80). Enasidenib (AG-221), mainly used to treat acute myeloid leukemia, exerts effects by binding to IDH2 mutants and blocking the production of 2-HG (99). However, the efficacy or safety of enasidenib has not been evaluated in T-FHCL.

### 4.4 Chimeric antigen receptor T-cell (CAR-T-cell) immunotherapy

In recent years, CAR-T-cell immunotherapy has demonstrated certain clinical benefits in hematologic tumors, targeting antigens with restricted expressions, such as CD1a, CD4, CD5, CD7, CD19, CD22, CD30, CD37, CCR4, TRBC1 and TRBC2 (100, 101).

However, in contrast to B-cell malignancies, in T-cell malignancies, CAR-T-cell immunotherapy has achieved more limited progress, owing to three major challenges: fratricide, T-cell aplasia, and product contamination with malignant T cells (101). Some solutions have been proposed to address these problems. Using alternative effector cells (such as NK cells) and genome editing approaches to reduce the expression of the CAR target antigen may be beneficial in preventing fratricide, which occurs when malignant and normal T cells express the same target antigen (101). In addition, utilizing CAR-T cells with a limited or adjustable lifespan or activity by adding safety switches may prevent T-cell aplasia (101). Furthermore, using allogeneic cells (such as multivirus-specific T cells and γδ T cells) as effector cells for CAR expression can effectively protect CAR−T-cell products from contamination with malignant T cells (101). Undoubtedly, until they are tested in the clinic, these CAR-T-cell immunotherapy alternatives cannot obtain optimum outcomes.

In a mouse model, CD30 CAR-T cells carrying three kinds of CD30 lentiviral CARs exhibited efficient cytotoxic effects on PTCL xenograft tumors, indicating that CD30 CAR-T-cell immunotherapy may be promising for cancer treatment (100). In a phase I dose escalation study (NCT03081910), CD5 CAR-T-cell therapy obtained an ORR of 44.4% (4/9), with a patient with AITL achieving CR (81). Recently, in a pioneering first-in-human phase I trial (NCT04572308), naturally selected CD7 CAR-T-cell immunotherapy was applied to 20 patients with R/R T-cell acute lymphoblastic leukemia (T-ALL, n=14) and lymphoblastic lymphoma (T-LBL, n=6). Nineteen patients achieved minimal residual disease-negative CR in the bone marrow by Day 28, and 5 of 9 patients achieved extramedullary CR (82).

Notably, several severe toxicities have been recorded for CAR-T-cell immunotherapy, including neurotoxicity, B-cell aplasia, cytokine release syndrome (CRS), and graft-versus-host disease (GVHD) (102). Such AEs of CAR-T-cell immunotherapy deserve attention and in-depth investigation. Numerous CAR-T-cell products for the treatment of PTCL are being investigated in preclinical and clinical trials. More data to validate the safety and efficacy of CAR-T-cell immunotherapy are urgently needed.

### 4.5 Other therapies

Lenalidomide, a thalidomide analog, exerts antiproliferative effects in cancer by repressing cereblon (CRBN) and angiogenesis and intensifying the immune response. Despite moderate single-agent activity, more promising results have been shown in combination regimens. However, in a phase II study (NCT01553786), the addition of lenalidomide to CHOP did not improve the complete metabolic response in previously untreated elderly AITL patients, and the most frequently recorded toxicities were hematologic (83). Another novel CRBN-binding agent, avadomide (CC-122), was evaluated in a phase I trial (NCT01421524) and achieved a 54% (7/13) ORR in advanced NHL, with only an AITL patient enrolled (86).

Antimetabolites are another common therapy for T-FHCL. Pralatrexate was the first drug approved in the USA to treat R/R PTCL, and it competitively inhibits dihydrofolate reductase (DHFR) (87, 103, 104). A single−arm, multicenter study (NCT0334933) recorded an ORR of 55% (11/20) in R/R AITL, with mucositis as the most common side effect (88). Forodesine, a novel purine nucleoside phosphorylase (PNP) inhibitor, has only been approved for the treatment of R/R PTCL in Japan (105). In the phase I/II study (NCT01776411), higher ORR values were reported in patients with R/R AITL (33%, 6/18) than in those with R/R PTCL-NOS (23%, 5/22) (89).

Farnesyltransferase (FTase) inhibitors have shown beneficial effects on Ras-transformed tumor cells. FTase inhibitors decrease RhoA and increase RhoC activity in breast cancer cells, which may be a clue for T-FHCL treatment (106). Tipifarnib, a potent and selective FTase inhibitor, was evaluated in a phase II study (NCT02464228), resulting in a 45.5% (5/11) ORR in R/R AITL (90). The prognosis appeared to be associated with KIR3DL2 and CXCL12 genotype (90).

Modulation of apoptosis is also a viable therapeutic strategy. The Bcl-2 inhibitor venetoclax showed single-agent activity in only 18% (3/17) of R/R PTCL patients, with one patient achieving CR (91). To enhance the rate of response, several combination regimens of venetoclax are being tested in trials (91). Treatment with the XPO1 inhibitor selinexor, which prevents the export of tumor suppressor proteins (TSPs) and reduces the expression of oncoproteins, including c-Myc, Bcl-2, and Bcl-6, resulted in 4 CRs and 18 PRs among 70 evaluable patients with R/R NHL (92). 2 patients with R/R PTCL were included and one of them achieved PR (92). Of note, selinexor combined with high-dose DICE (dexamethasone, ifosfamide, carboplatin, and etoposide) achieved a 100% (10/10) ORR in R/R TCL and natural killer/T-cell lymphoma but was poorly tolerated (93). There is insufficient data on the efficacy of MDM2 inhibitors in the treatment of T-FHCL.

## 5 Discussion and future perspectives

As the cellular origin of T-FHCL, Tfh cells differentiate based on IL-21, IL-6, and B cells. Tfh cell dysregulation is associated with the pathogenesis of several types of tumors, as evidenced by markers of Tfh cells (32). As a component of the lymphoma microenvironment, Tfh cells are associated with poor prognosis (32). Understanding the role of human Tfh cells in GC and cancer progression will provide new directions for novel immune strategies in multiple human cancers.

The histological diagnosis of T-FHCL is full of challenges. Polymorphous infiltration, low neoplastic cell content, and EBV-driven B-cell proliferation may contribute to misdiagnosis (107). Hence, assessment of the molecular pathology of T-FHCL must be considered an auxiliary diagnostic strategy. The MICM (Morphology, Immunology, Cytogenetics, and Molecular Biology) diagnostic model, which has been applied to leukemia classification, can significantly improve the accuracy of T-FHCL diagnosis, paving the way for targeted therapies. The immune microenvironment in PTCL is profoundly immunosuppressive. CD4+ T cell- and lymphodepletion and the recruitment of tumor-associated macrophages are relevant significant causes, which may be predictive of chemotherapy and immunotherapy outcomes for PTCL patients (45, 108). However, there is rare research to provide sufficient evidence. Of particular note, molecular mutation analysis seems to be a promising method for the early detection of T-FHCL (107). Furthermore, comprehensive mutation analysis may serve as a more sensitive biomarker for predicting response and estimating the vulnerability to targeted therapy in patients with PTCL and its subtypes, which has been practiced in several trials (78, 79). In recent years, emerging techniques such as single-cell sequencing and spatial transcriptomics have deepened research and refined the identification of T-FHCL at the molecular level.

Because of the limited efficacy of the CHOP regimen as first-line therapy in T-FHCL, novel agents or regimens are urgently needed to improve prognosis. Resistance to first-line regimens is found associated with specific mutations. The resistance of anthracycline treatment in AITL was attribute to DNMT3A R882X mutation, which contributes to impairing nucleosome eviction and chromatin remodeling (83). Non–anthracycline-based frontline chemotherapy or novel combinations may be a potent way to overcome the resistance. Besides, perturbation of TP53 seem be a significant determinant of frontline therapy resistance in PTCL (109). T-FHCL is characterized by epigenetic disruption and thus has a unique vulnerability to epigenetic inhibitors, which have demonstrated marked single-drug activity. Furthermore, combination regimens that rely on the synergistic effects of novel agents are being explored. For example, romidepsin has been combined with duvelisib (NCT02783625), pembrolizumab (NCT03278782), 5-azacitidine (NCT01998035), and CHOP (NCT01796002) for the treatment of PTCL (46, 70, 78). Although the first three regimens showed satisfactory safety, tolerability, and clinical benefits, only the Ro-CHOP regimen was evaluated in large cohorts of patients, though it did not prolong PFS and had higher toxicity (46, 70, 78). Feasible epigenetic inhibitor combination regimens warrant further exploration and investigation.

CAR-T-cell immunotherapy and HSCT have exhibited excellent clinical benefits for hematologic malignancies, increasing the limited number of treatment options for T-FHCL. Despite the emergence of new targeted agents, HSCT has been consistently employed, while targeted therapies offer a safety guarantee in HSCT pretreatment, maintenance therapy, and salvage therapy. The combination of novel targeted agents and HSCT may be a promising strategy for T-FHCL treatment.

Because of the low incidence of T-FHCL, it is difficult to recruit enough patients for clinical trials through international registries, resulting in a lack of experience. Therapeutic options for T-FHCL are still limited, representing an unmet clinical need. More effective and safe targeted agents and combination regimens should be explored *via* multicenter international efforts and applied in the clinic to confirm their therapeutic potential. Furthermore, identifying correlations between unique mutation profiles and drug responses is the key to tailoring individualized strategies for each T-FHCL patient.

## Author contributions

JD designed this study and guided the entire process. SJ, LZ, and ZC wrote the manuscript. SJ prepared **Tables 1**–**6**. SJ, MZ, and WL prepared **Figures 1**–**4**. JD and XF edited the original draft, tables, and figures. JD, WL, ZH, JY, JH, and TW supervised the project and ultimately approved the manuscript. All authors contributed to the article and approved the submitted version.

## Glossary

**Table:**

T-FHCL	nodal T-follicular helper cell lymphoma
PTCL	peripheral T-cell lymphoma
NHL	non-Hodgkin lymphoma
Tfh	T-follicular helper
Th	T helper
GC	germinal center
AITL	angioimmunoblastic T-cell lymphoma
FTCL	follicular T-cell lymphoma
NPTCL-TFH	nodal peripheral T-cell lymphoma with T-follicular helper phenotype
R/R	relapsed/refractory
DLBCL	diffuse large B-cell lymphoma
T-NHL	T-cell non-Hodgkin lymphoma
ATLL	adult T-cell leukemia/lymphoma
OS	overall survival
ORR	overall response rate
CR	complete response
CRR	complete response rate
CR/CRu	confirmed/unconfirmed complete response
PR	partial response
PFS	progression-free survival
HSCT	hematopoietic stem cell transplantation
auto-SCT	autologous stem cell transplantation
allo-SCT	allogeneic stem cell transplantation
FDC	follicular dendritic cell
HEV	high endothelial venule
EBV	Epstein‒Barr virus
CD	cluster of differentiation
DNMT	DNA methyltransferase
CCR4	chemokine receptor 4
HDAC	histone deacetylase
ICOS	inducible T-cell costimulator
mTOR	mammalian target of rapamycin
PD-1	programmed death receptor 1
PI3K	phosphoinositide 3-kinase
JAK	Janus kinase
SYK	spleen tyrosine kinase
ITK	IL-2 inducible T-cell kinase
ERK	extracellular regulated protein kinase
STAT	signal transducer and activator of transcription
NF-κB	nuclear factor kappa-B
AURKA	Aurora A kinase
XPO1	exportin 1
TRBC1	T-cell receptor β-chain constant domain 1
CRBN	cereblon
IKZF	Ikaros zinc finger
CRL4	Cullin4-RING ligase
TCR	T-cell receptor
RHOA	ras homolog member A
TET2	tet methylcytosine dioxygenase 2
DNMT3A	DNA methyltransferase 3 alpha
IDH2	isocitrate dehydrogenase 2
GTP	guanine nucleotide triphosphate
GAP	GTPase-activating protein
GEF	guanine nucleotide exchange factor
GDI	guanine nucleotide dissociation inhibitor
VAV1	vav guanine nucleotide exchange factor 1
MAPK	mitogen-activated protein kinase
PKC	protein kinase C
PTK	protein tyrosine kinase
PLCγ1	phospholipase C gamma 1
NFAT	nuclear factor of activated T cells
DHFR	dihydrofolate reductase
PNP	purine nucleoside phosphorylase
FTase	farnesyltransferase
MDM2	murine double minute 2
cIAP	cellular inhibitor of apoptosis protein
XIAP	X-linked inhibitor of apoptosis protein
CAR-T-cell	chimeric antigen receptor T-cell
CHOP	cyclophosphamide, doxorubicin, vincristine, and prednisone
CHP	cyclophosphamide, doxorubicin, and prednisone
CHOEP	cyclophosphamide, doxorubicin, vincristine, etoposide, and prednisone
DICE	dexamethasone, ifosfamide, carboplatin, and etoposide
NGS	next-generation sequencing
MICM	morphology, immunology, cytogenetics, and molecular biology
